# Effects of *Clostridium beijerinckii* and Medium Modifications on Acetone–Butanol–Ethanol Production From Switchgrass

**DOI:** 10.3389/fbioe.2022.942701

**Published:** 2022-08-03

**Authors:** Tinuola Olorunsogbon, Yinka Adesanya, Hasan K. Atiyeh, Christopher Chukwudi Okonkwo, Victor Chinomso Ujor, Thaddeus Chukwuemeka Ezeji

**Affiliations:** ^1^ Department of Animal Science, The Ohio State University, Wooster, OH, United States; ^2^ Biosystems and Agricultural Engineering, Oklahoma State University, Stillwater, OK, United States; ^3^ Biotechnology Program, College of Science, The Roux Institute, Northeastern University, Portland, ME, United States; ^4^ Department of Food Science, University of Wisconsin-Madison, Maddison, WI, United States

**Keywords:** ABE fermentation, ammonium carbonate, non-detoxified switchgrass hydrolysate, LDMIC, short-chain dehydrogenase/reductase, *Clostridium beijerinckii*

## Abstract

The presence of lignocellulose-derived microbial inhibitory compounds (LDMICs) in lignocellulosic biomass (LB) hydrolysates is a barrier to efficient conversion of LB hydrolysates to fuels and chemicals by fermenting microorganisms. Results from this study provide convincing evidence regarding the effectiveness of metabolically engineered *C. beijerinckii* NCIMB 8052 for the fermentation of LB-derived hydrolysates to acetone–butanol–ethanol (ABE). The engineered microbial strain (*C. beijerinckii*_SDR) was produced by the integration of an additional copy of a short-chain dehydrogenase/reductase (SDR) gene (*Cbei_*3904) into the chromosome of *C. beijerinckii* NCIMB 8052 wildtype, where it is controlled by the constitutive thiolase promoter. The *C. beijerinckii*_SDR and *C. beijerinckii* NCIMB 8052 wildtype were used for comparative fermentation of non-detoxified and detoxified hydrothermolysis-pretreated switchgrass hydrolysates (SHs) with and without (NH_4_)_2_CO_3_ supplementation. In the absence of (NH_4_)_2_CO_3_, fermentation of non-detoxified SH with *C. beijerinckii*_SDR resulted in the production of 3.13- and 2.25-fold greater quantities of butanol (11.21 g/L) and total ABE (20.24 g/L), respectively, than the 3.58 g/L butanol and 8.98 g/L ABE produced by *C. beijerinckii*_wildtype. When the non-detoxified SH was supplemented with (NH_4_)_2_CO_3_, concentrations were similar for butanol (9.5 compared with 9.2 g/L) and ABE (14.2 compared with 13.5 g/L) produced by *C. beijerinckii*_SDR and *C. beijerinckii*_wildtype, respectively. Furthermore, when *C. beijerinckii*_SDR and *C. beijerinckii*_wildtype were cultured in detoxified SH medium, *C. beijerinckii*_SDR produced 1.11- and 1.18-fold greater quantities of butanol and ABE, respectively, than when there was culturing with *C. beijerinckii*_wildtype. When the combined results of the present study are considered, conclusions are that the microbial strain and medium modifications of the fermentation milieu resulted in greater production of fuels and chemicals from non-detoxified LB hydrolysates.

## 1 Introduction

*Clostridium* species can ferment a wide variety of substrates such as starch, disaccharides, hexoses, pentoses, glycerol, cellulose, and syngas into industrially important chemicals and environmentally compatible fuels (butanol, ethanol, isopropanol, and hexanol; Ezeji et al., 2007a; Phillips et al., 2015; Liu et al., 2015; Sandoval-Espinola et al., 2017; Sun et al., 2019). Solventogenic *Clostridium* species exhibit a biphasic fermentation pattern characterized by acid (acetic acid and butyric acid) production in the exponential phase (acidogenesis) and solvent production (acetone, butanol, and ethanol; solventogenesis) in the second (stationary phase). During acidogenesis, there is the production of H_2_, CO_2_, acetate, and butyrate, resulting in a decrease in culture pH. During solventogenesis, there is a marked change in metabolism resulting in the uptake of acids produced during the acidogenic phase along with sugar substrates, which are metabolized into acetone, butanol, and ethanol (ABE; Jones and Woods, 1986; Veza, et al., 2021). A typical batch fermentation for producing ABE using *Clostridium* species results in the molar ratio of 3:6:1 for acetone, butanol, and ethanol, respectively.

Butanol or ABE is currently produced at a laboratory scale by fermenting food crops such as corn and sugarcane, which raises concerns over human food security. Consequently, exploring non-food substrates—lignocellulosic biomass (LB) such as energy crops, agricultural residues, and farm wastes—for biofuel production is being considered a “panacea” for preventing or reducing prospective competition between food sources and industrial raw materials for biofuels production (Palmqvist and Hahn-Hagerdal, 2000; Ezeji et al., 2007a; Isar and Rangaswamy, 2012; Zhang and Ezeji, 2013; Okonkwo et al., 2016; Sodre, et al., 2021). LB is composed of polymeric sugar forms (cellulose and hemicellulose) and lignin. The compact nature of LB, however, makes it recalcitrant to enzymatic hydrolysis to release fermentable monomeric sugars (glucose, xylose, and arabinose). Hence, there is need for a pretreatment process prior to enzyme-mediated hydrolysis, and this typically involves the application of heat and acid/alkaline to facilitate the breakdown of lignin matrix of LB. Nonetheless, in addition to releasing fermentable sugars, the pretreatment process releases acetate from the hemicellulose component of LB alongside the generation of a plethora of toxic phenolic and furanic aldehydes generally referred to as lignocellulose-derived microbial inhibitory compounds (LDMICs; Ezeji et al., 2007b; Veza, et al., 2021; Sodre, et al., 2021). LDMICs such as furanic aldehydes (e.g., furfural and hydroxymethylfurfural-HMF) and phenolics (hydroxybenzaldehyde, ferulic acid, and syringic acid, etc.) impair the growth and capacity of fermenting microorganisms to utilize sugars thereby resulting in low yield of biofuels (Ezeji et al., 2007b; Ezeji and Blaschek 2008; Baral and Shah, 2014; Ujor et al., 2016). The presence of LDMICs in hydrolysates, therefore, is a major impediment to the use of LB for biofuel production. Various researchers have explored LB hydrolysates (LBH) detoxification processes such as overliming (Qureshi et al., 2010; Zhang et al., 2018), use of activated carbon (Liu et al., 2015; Dong et al., 2018), and media optimization strategies such as glycerol (Ujor et al., 2014) and allopurinol (Ujor et al., 2015) supplementations, to mitigate the inhibitory effects of LDMICs on fermenting microorganisms. These strategies, however, have associated costs, therefore, leading to relatively greater biofuel production costs (Liu et al., 2015). Intermittent addition of substrates during fermentation has been explored as means of circumventing LDMICs-mediated toxicity. With this strategy, there was enhanced bioconversion of LBH to butanol, thereby raising the prospect of eliminating the need for detoxification of hydrothermolysis pretreated LBH prior to fermentation (Adesanya et al., 2022).

To further reduce or eliminate cost associated with detoxification of LBH prior to fermentation, metabolic engineering of solventogenic *Clostridium* species for increased tolerance to LDMICs is viewed as a possible strategy to markedly improve efficacy and efficiency, and consequently economics of fermenting undetoxified LBH into butanol (Agu et al., 2019). Toward this goal, a LDMIC-tolerant strain of *C. beijerinckii* was engineered to overexpress a short-chain dehydrogenase/reductase (SDR) (Okonkwo et al., 2019). This approach was based on results from a previous genome-wide transcriptional study that showed significant upregulation of the open reading frame *Cbei_*3904 in *C. beijerinckii*, which encodes a NAD(P)H-dependent SDR when furfural was supplemented in the growth medium (Zhang and Ezeji, 2013). The SDR encoded by *Cbei_*3904 has been shown to be involved in the transformation of furfural and HMF to their respective less inhibitory alcohols (furfuryl alcohol and 2,5-bis-hydroxymethylfuran-HMF alcohol) in *C. beijerinckii* (Zhang et al., 2015). The resulting metabolically engineered strain of *C. beijerinckii* (*C. beijerinckii*_SDR) however, has not been evaluated for tolerance to LDMICs in undetoxified LBH. The present study, therefore, was conducted to evaluate the capacity of *C. beijerinckii*_SDR to ferment undetoxified switchgrass hydrolysates (SH) to butanol.

## 2 Materials and Methods

### 2.1 Production of Switchgrass Hydrolysates

Dried *Panicum virgatum L.* (Alamo switchgrass) was collected from the Gasification llaboratory at Oklahoma State University and processed using a hammer mill equipped with a 2 mm sieve. The comminuted switchgrass was pretreated in a 1 L Parr reactor (Parr series 4520, Parr instrument company, Moline IL, United States) at a loading rate of 10% solids at 200°C for 10 min (Pessani et al., 2011; Liu et al., 2015). After cooling, the pretreated switchgrass was vacuum filtered with Whatman no. 4 filter paper to separate solids from the liquid component containing mainly degraded hemicellulose (xylose), according to a previously described method (Adesanya et al., 2022). The solids were washed four times with deionized water and enzymatically hydrolyzed using Accellerase 1500 (gifted by DuPont, Rochester, NY, United States) in a shaker water bath, at 50°C and 250 rpm as described previously (Liu et al., 2015; Adesanya et al., 2022).

### 2.2 Detoxification of Hydrolyzed Switchgrass Hydrolysates

A portion of the SH was detoxified using activated carbon to reduce the concentrations of LDMICs produced during the pretreatment. Detoxification was conducted with Calgon rod-shaped activated carbon (AP4-60, Calgon Carbon Corporation, Pittsburgh, PA) using the treatment regimen described by Adesanya et al. (2022).

### 2.3 Bacterial Strains and Culture Conditions

*Clostridium beijerinckii* NCIMB 8052 was purchased from the American Type Culture Collection (Manassas, VA, United States) as *C. beijerinckii* ATCC 51743. In an earlier study, *C. beijerinckii*_SDR was constructed by integrating the open reading frame *Cbei*_3904 (which encodes an SDR) into the chromosome of *C. beijerinckii* NCIMB 8052 to obtain the LDMIC-tolerant *C. beijerinckii*_SDR (Okonkwo et al., 2019). The chromosomally integrated *SDR* gene was constitutively expressed by placing it under the control of thiolase promoter from *C. acetobutylicum* ATC 824. *Clostridium* strains (*C. beijerinckii*_SDR and *C. beijerinckii* NCIMB 8052) were stored as spore suspensions in ddH_2_O at 4°C (Han et al., 2013). Spores for each strain (400 µl) were reactivated using heat shock treatments at 75°C for 3 min prior to inoculation of 10 ml anoxic Tryptone-Glucose-Yeast extracts (TGY) medium (Ezeji et al., 2013; Liu et al., 2015). The culture medium was incubated at 35°C overnight (12–13 h) during which OD_600 nm_ of 0.9–1.1 was attained. Approximately 2 ml of an overnight culture of each strain (10%, v/v) was used to inoculate 18 ml of fresh anoxic TGY medium to increase the quantity of the preculture. These new cultures were incubated at 35°C until OD ∼ 0.9 to 1.1 was attained (3–4 h), after which they were used to inoculate the fermentation medium. All inoculations, handling, and incubation processes were performed in an anaerobic chamber (Coy Laboratory Products Inc., Grass Lake, MI) with a modified atmosphere of 82% N_2_, 15% CO_2_, and 3% H_2._ The TGY medium in loosely capped bottles and test tubes was maintained overnight in the anaerobic chamber to allow for the removal of residual oxygen before fermentation was initiated (Ezeji et al., 2013; Liu et al., 2015; Sun et al., 2020).

### 2.4 Acetone–Butanol–Ethanol Fermentation of Non-Detoxified Switchgrass Hydrolysates

Fermentation was conducted in 150-ml Pyrex screw-cap bottles with a 50 ml working volume. To initiate fermentation, non-detoxified SH (44.5 ml) was transferred into pre-sterilized 150-ml screw-cap bottles which were then supplemented with 1% (v/v) of each of the P2 buffer, mineral, and vitamin stock solutions (Table 1; Ezeji et al., 2003). Sterile yeast extract (1 ml of 50 g/L stock) was aseptically added to the non-detoxified SH fermentation medium followed by the addition of 3 ml (6%, v/v) of *C. beijerinckii*_SDR or *C. beijerinckii*_wildtype preculture.

**TABLE 1:** Compositions of fermentation and preculture media and stock solutions used in the fermentation of SH by *Clostridium beijerinckii*.

Medium component	Formula	Amount g/L
Switchgrass hydrolysates		
Non-detoxified SH glucose		61.2
Detoxified SH hydrolysates glucose		58.7
Yeast extract		1
Glucose P2 medium		
Glucose	C_6_H_12_O_6_	60
Yeast extract	—	1
TGY medium		
Tryptone	—	30
Glucose	C_6_H_12_O_6_	20
Yeast extract	—	10
L-Cysteine	C_3_H_7_NO_2_S	1
P2 buffer stock solution		
Potassium phosphate monobasic	KH_2_PO_4_	50
Potassium phosphate dibasic	K_2_HPO_4_	50
Ammonium acetate	NH_4_CH_3_CO_2_	220
Adjusted P2 buffer stock solution		
Potassium phosphate monobasic	KH_2_PO_4_	50
Potassium phosphate dibasic	K_2_HPO_4_	50
Ammonium acetate	NH_4_CH_3_CO_2_	50
Vitamins		
p-(4)-Aminobenzoic acid	C_7_H_7_NO_2_	0.1
Thiamine	C_12_H_17_N_4_OS^+^	0.1
Biotin	C_10_H_16_N_2_O_3_S	0.01
Mineral stock solution		
Magnesium sulfate heptahydrate	MgSO_4_·7H_2_O	20
Manganese sulfate heptahydrate	MnSO_4_·7H_2_O	1
Ferrous sulfate heptahydrate	FeSO_4_·7H_2_O	1
Sodium chloride	NaCl	1
Supplement		
Yeast extract stock		50
Ammonium carbonate stock	(NH_4_)_2_CO_3_	200

For fermentations using the modified medium [with (NH_4_)_2_CO_3_ supplementation], 44.0 ml of non-detoxified SH was transferred into another set of 150-ml Pyrex screw-cap bottles. Modified P2 buffer, mineral, and vitamin stock solutions (1%, v/v each) were added (Table 1). Yeast extract (1 ml of 50 g/L stock) was added to the medium containing the non-detoxified SH resulting in a 1 g/L final concentration. Also, (NH_4_)_2_CO_3_ (500 µl of 200 g/L stock) was added resulting in a final concentration of 2 g/L in the fermentation medium.

### 2.5 Fermentation of Detoxified Switchgrass Hydrolysates to Acetone–Butanol–Ethanol

Fermentation was conducted in 150-ml Pyrex screw-cap bottles containing a 50 ml working volume of fermentation medium. *C. beijerinckii*_SDR and *C. beijerinckii*_wildtype (6%, v/v) were separately inoculated into the detoxified SH fermentation medium as described previously for the non-detoxified SH fermentation medium. As with the undetoxified SH medium, another set of fermentation cultures was established with the addition of (NH_4_)_2_CO_3_ to a final concentration of 2 g/L. P2 medium (60 g/L glucose; 1 g/L yeast extract) was used as a positive control. For the positive control, 45.5 ml of P2 medium was transferred into another set of 150-ml Pyrex screw-cap bottles. Subsequently, the P2 buffer, mineral, and vitamin stock solutions (1%, v/v each) were added using the methods described previously. The compositions of the P2 buffer, minerals, and stock solutions are presented in Table 1. All fermentation experiments were conducted in triplicate. Samples (1 ml) were collected from each bottle at the time of initiation of the fermentation (0 h) and subsequently at 12 h intervals for 72–120 h to determine pH, OD_600 nm_, sugar, butanol, acetone, ethanol, acetic, and butyric acid concentrations.

### 2.6 Analytical Methods

Optical density was determined at 600 nm using a DU^®^ 800 spectrophotometer (Beckman Coulter Inc., Brea, CA, United States) to estimate the changes in the growth of the *C. beijerinckii* strains during fermentation. Gas chromatography was conducted to quantify the concentrations of acetone, butanol, ethanol, acetic, and butyric acids using a 7890A system (Agilent Technologies 7890, Agilent Technologies Inc., Wilmington, DE), according to a previously described method (Ujor et al., 2021). Sugar concentrations (glucose, xylose, and arabinose) were analyzed using HPLC (Waters, Milford, MA, United States) equipped with an evaporative light scattering detector (Waters, Milford, MA, United States) according to a previous method (Ujor et al., 2021). The concentrations of the LDMICs were quantified using HPLC according to the method of Agu et al., 2016. The ABE yield was determined by dividing the total grams of ABE produced by the total grams of glucose or sugars utilized during fermentation, while ABE productivity was calculated by dividing the maximum amount of ABE (g/L) produced by the corresponding fermentation time (h). The Glucose utilization rate was calculated by dividing the total concentration of glucose used to produce the maximum ABE by the corresponding fermentation time.

### 2.7 Statistical Analyses

A one-way ANOVA and student’s t-test analyses were performed using the general linear model (GLM) procedure of SAS OnDemand for Academics 3.1.0 (SAS Institute Inc., Cary, NC). The least square difference (LSD) test procedure was used for evaluation of treatment means. There was the determination of values for maximum optical density readings, sugar utilization during the fermentation periods, as well as maximum concentrations, yield, and production of butanol and ABE. Mean differences in these values because of using different *C. beijerinckii* strains and/or different fermentation medium compositions were tested for significance when there was a *p* ≥ 0.05.

## 3 Results

### 3.1 Production and Detoxification of Switchgrass Hydrolysates

The hydrothermolysis pretreatment method was efficacious for deconstructing switchgrass biomass. The composition of the pretreated solids has been reported by Adesanya et al. (2022). Two batches of SH were utilized. The SHs were subjected to enzymatic hydrolysis to produce fermentable monomeric sugars. After hydrolysis, Batch 1 of the SH was detoxified using activated carbon to remove/reduce LDMICs while the SH in Batch 2 was not detoxified. Glucose concentrations of Batches 1 (detoxified) and 2 (non-detoxified) of the SH were 58.7 and 61.2 g/L, respectively (Table 2). The concentrations of LDMICs in the medium containing detoxified and non-detoxified SH are presented in Table 2. In the medium based on the detoxified SH, the concentrations of furfural, HMF, vanillic acid, syringic acid, p-coumaric acid, and hydroxybenzaldehyde were 2-, 1.06-, 3.7-, 6.9-, 1.8-, and 1.6-fold lower, respectively, when compared to the concentrations in the non-detoxified SH medium.

**TABLE 2:** Concentrations of glucose and LDMICs in detoxified and non-detoxified SH. Standard deviation for glucose reading represents duplicate (*n* = 2).

Compound	Non-detoxified SH (g/L)	Detoxified SH (g/L)
Sugar		
Glucose	61.2 ± 0.29	58.7 ± 0.74
LDMICs	mg/L	mg/L
HMF	9.52	8.95
Furfural	6.03	2.93
Vanillic acid	2.20	0.59
Syringic acid	6.15	0.89
Coumaric acid	1.06	0.59
Hydroxybenzaldehyde	2.92	1.88

### 3.2 Fermentation Profiles of the *C. beijerinckii* Strains During Growth in Non-Detoxified and Detoxified Switchgrass Hydrolysates

#### 3.2.1 The Growth Profiles of *C. beijerinckii*_wildtype and *C. beijerinckii*_SDR

**FIGURE 1:**
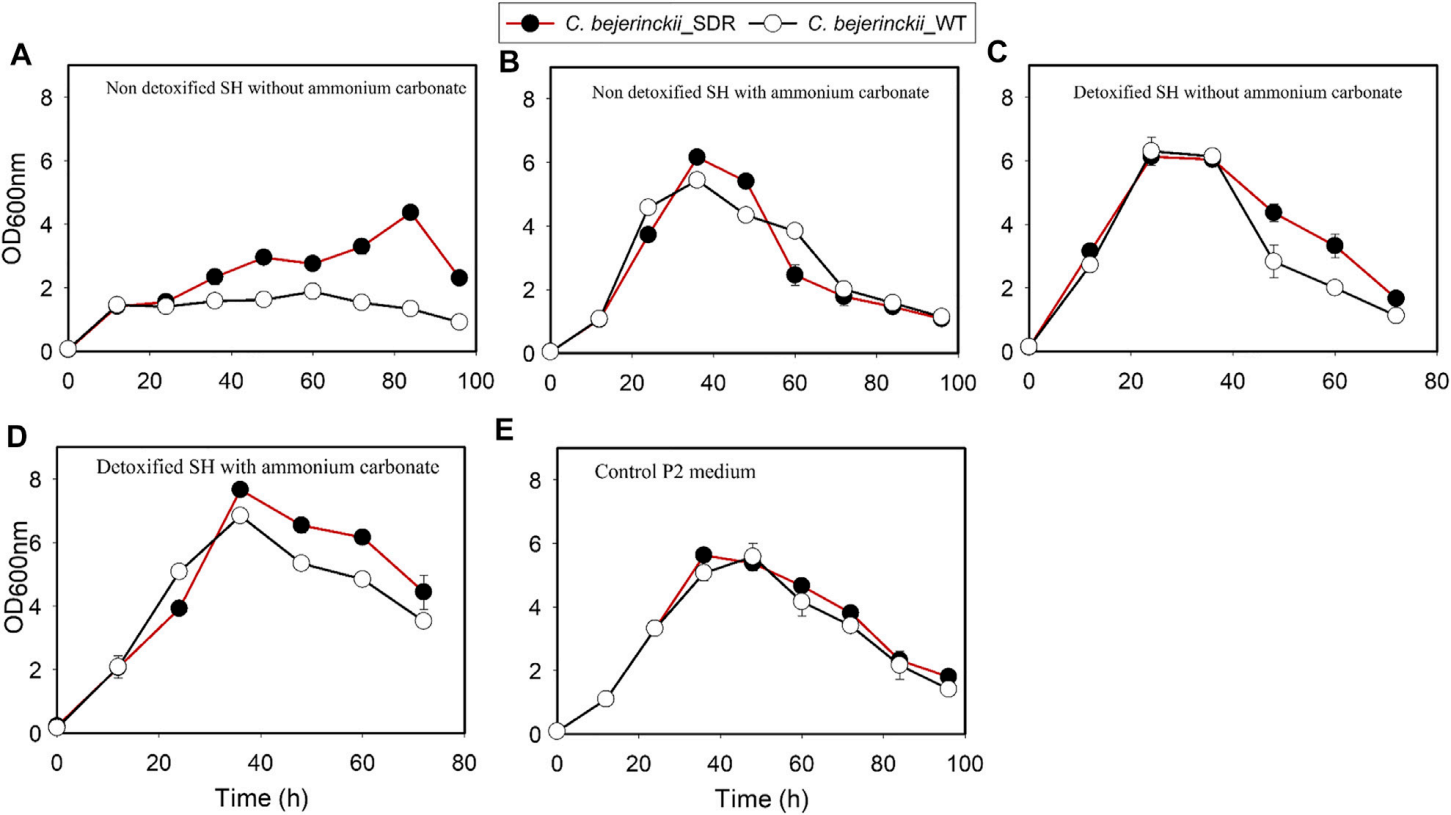
Estimated population profiles for *C. beijerinckii*_SDR and *C. beijerinckii*_wildtype during fermentation of SH and P2 medium control. **(A)** Non-detoxified SH without ammonium carbonate. **(B)** Non-detoxified SH with ammonium carbonate. **(C)** Detoxified SH without ammonium carbonate. **(D)** Detoxified SH with ammonium carbonate. **(E)** Control P2 medium.

The increase in growth by *C. beijerinckii*_wildtype and *C. beijerinckii*_SDR was rapid, attaining maximum OD_600 nm_ of 5.7 and 5.9, respectively, in the glucose P2 medium (positive control; Figure 1). Similarly, there was a marked increase in growth for both *C. beijerinckii*_wildtype and *C. beijerinckii*_SDR, reaching maximum OD_600 nm_ of 6.3 and 6.1, respectively, in the medium containing the detoxified SH (Figure 1). Similarly, when the detoxified SH medium was supplemented with (NH_4_)_2_CO_3_, there was a discernible rapid increase in the growth of *C. beijerinckii*_wildtype and *C. beijerinckii*_SDR, attaining a maximum OD_600 nm_ of 6.8 and 7.7, respectively (Figure 1). Conversely, when *C. beijerinckii*_wildtype and *C. beijerinckii*_SDR were cultured in a non-detoxified SH medium, there was reduced growth for both strains, attaining maximum OD_600 nm_ of 1.9 and 4.4, respectively, (Figure 1). As expected, there was an increase in population size for *C. beijerinckii*_wildtype and *C. beijerinckii*_SDR during fermentation in non-detoxified SH medium supplemented with (NH_4_)_2_CO_3_ reaching OD_600 nm_ of 5.4 and 6.2, respectively (Figure 1).

#### 3.2.2 pH and Acid Concentrations in the Fermentation Cultures of *C. beijerinckii*_wildtype and *C. beijerinckii*_SDR

**FIGURE 2:**
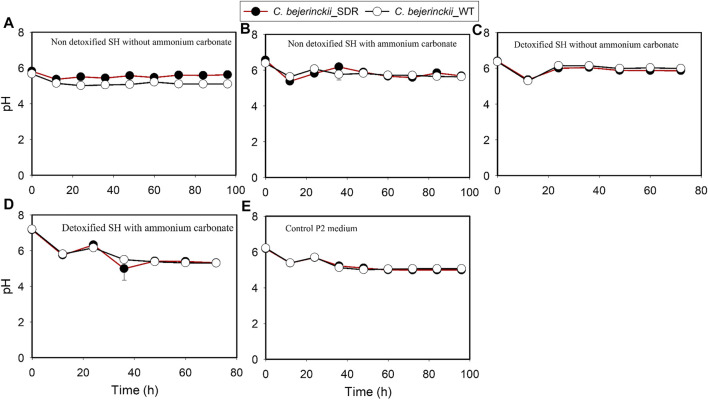
pH profiles of the culture medium during fermentation of SH and P2 medium control by *C. beijerinckii*_SDR or *C. beijerinckii*_wildtype. **(A)** Non-detoxified SH without ammonium carbonate. **(B)** Non-detoxified SH with ammonium carbonate. **(C)** Detoxified SH without ammonium carbonate. **(D)** Detoxified SH with ammonium carbonate. **(E)** Control P2 medium.

**FIGURE 3:**
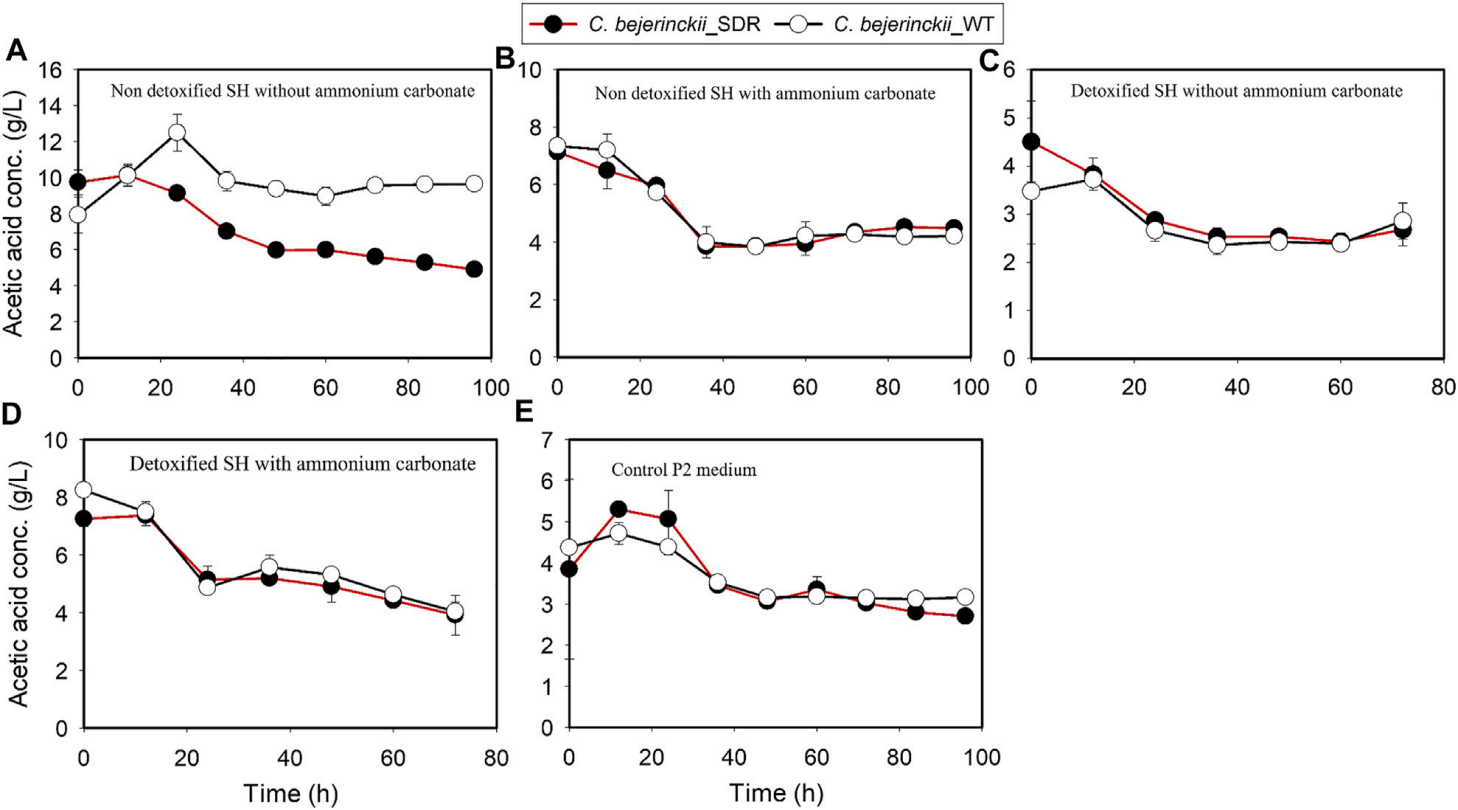
Acetic acid concentration profiles in the fermentation media using *C. beijerinckii*_SDR or *C. beijerinckii*_wildtype. **(A)** Non-detoxified SH without ammonium carbonate. **(B)** Non-detoxified SH with ammonium carbonate. **(C)** Detoxified SH without ammonium carbonate. **(D)** Detoxified SH with ammonium carbonate. **(E)** Control P2 medium.

**FIGURE 4:**
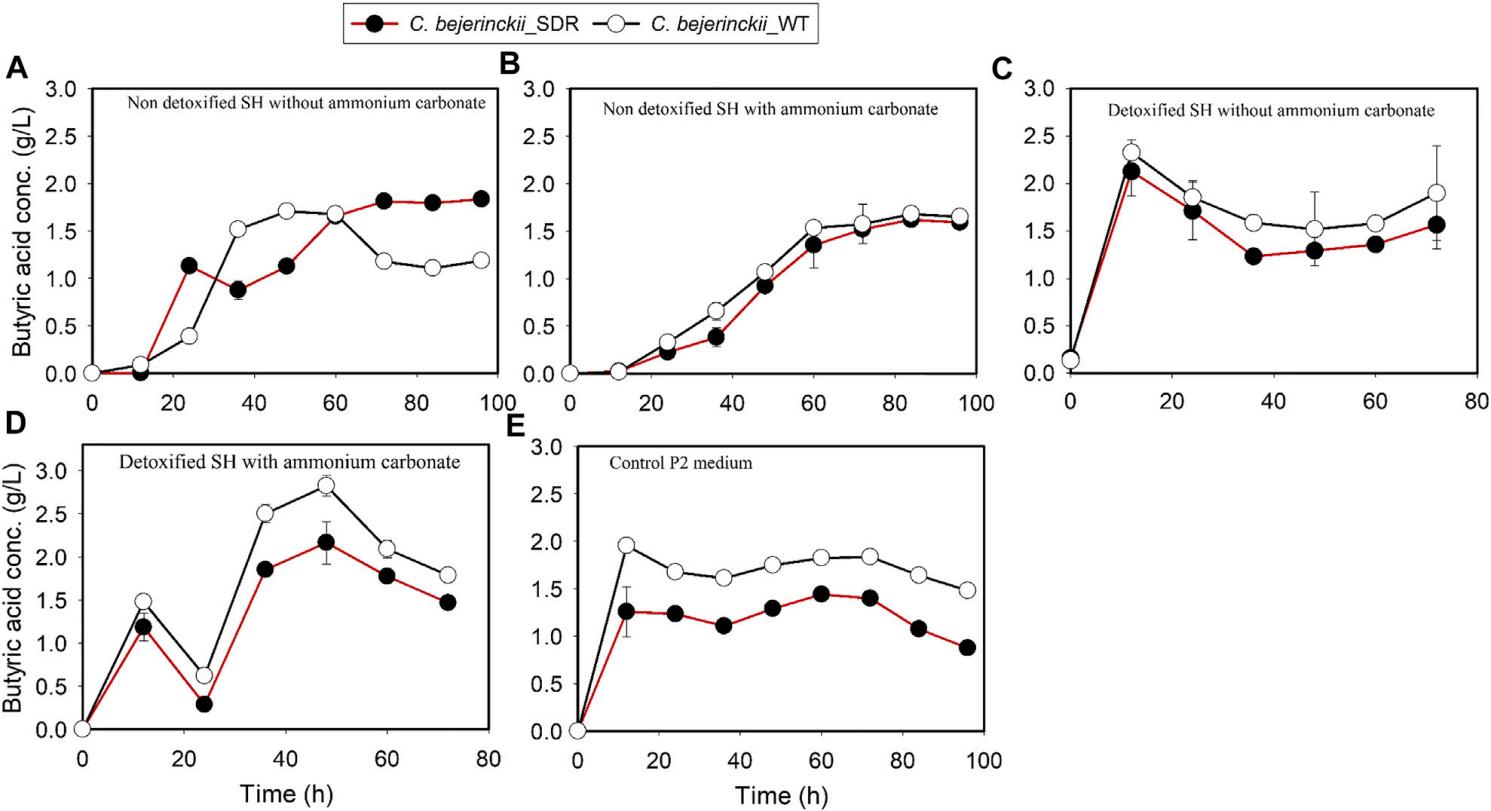
Butyric acid concentration profiles in the fermentation media using *C. beijerinckii*_SDR or *C. beijerinckii*_wildtype. **(A)** Non-detoxified SH without ammonium carbonate. **(B)** Non-detoxified SH with ammonium carbonate. **(C)** Detoxified SH without ammonium carbonate. **(D)** Detoxified SH with ammonium carbonate. **(E)** Control P2 medium.

The pH, acetic acid, and butyric acid values are depicted in Figures 2–4 for cultures of *C. beijerinckii* NCIMB 8052 and *C. beijerinckii*_SDR grown in non-detoxified and detoxified SH. As expected, during fermentation of non-detoxified SH without (NH_4_)_2_CO_3_ supplementation, the pH of the fermentation medium decreased to less than 5.4 (Figure 2A) at 12 h with a concomitant increase in acetic (Figure 3A) and butyric acids concentrations (Figure 4A). While the pH increased after 12 h with *C. beijerinckii*_SDR, the pH did not increase significantly in cultures inoculated with *C. beijerinckii*_wildtype (Figure 2A). Meanwhile, when non-detoxified SH medium was supplemented with (NH_4_)_2_CO_3_, the pH for both *C. beijerinckii*_wildtype and *C. beijerinckii*_SDR increased after 12 h of fermentation (Figure 2B) with a concomitant decrease in acetic acid concentration (Figure 3A), a trend that is similar to the pH of the P2 medium control (Figures 2E, 3E, 4E). Notably, the butyric acid concentration remained relatively small in the fermentation media (Figures 4A,B). To determine the extent to which the resulting pH and acid profiles of *C. beijerinckii*_wildtype during fermentation of non-detoxified SH negatively affected the cells (in addition to the effects of the LDMICs), the SH was detoxified by utilizing activated carbon to reduce the concentrations of LDMICs (Table 2). The pH and acid profiles of *C. beijerinckii*_wildtype and *C. beijerinckii*_SDR improved markedly during fermentation of detoxified SH and showed the typical pH profile of ABE fermentation—pH decreases before and increases after 12 h of fermentation (Figure 2C). Furthermore, prior to 12 and after 12 h of fermentation, the observed decrease and increase in culture pH coincided with an increase and decrease in butyric acid concentration, respectively (Figure 4C). The observed fluctuations in culture pH and acid concentration are due to acid production and re-assimilation of acidic constituents by *C. beijerinckii*_wildtype and *C. beijerinckii*_SDR, typical of what occurs with the exponential growth/acidogenic, and solventogenic phases of ABE fermentation, which indicate a relatively good physiological state of the culture during growth in detoxified SH or non-detoxified SH with (NH_4_)_2_CO_3_ supplementation. Similarly, when *C. beijerinckii*_wildtype and *C. beijerinckii*_SDR were grown in detoxified SH medium supplemented with (NH_4_)_2_CO_3_, the pH of the cultures decreased and later increased as fermentation progressed (Figure 2D). Conversely, at the same fermentation time points, the concentration of acids increased which later decreased as fermentation progressed (Figures 3D, 4D). Notably, the acetic acid content of the fermentation medium at 0 h (Figure 3) was from ammonium acetate, which is contained in the P2 buffer.

#### 3.2.3 Sugar Utilization During Fermentation in Switchgrass Hydrolysates

Most of the pentose content of the SH after pretreatment by hydrothermolysis was recovered during the washing process leaving mostly cellulose, which was later hydrolyzed to glucose using a commercial cellulase. Table 3 shows, the total glucose uptake by the LDMICs-tolerant *C. beijerinckii*_SDR and *C. beijerinckii*_wildtype during the fermentation of non-detoxified SH, non-detoxified SH with (NH_4_)_2_CO_3_ supplementation, detoxified SH, and detoxified SH with (NH_4_)_2_CO_3_ supplementation. While the LDMIC-tolerant *C. beijerinckii*_SDR readily utilized the glucose in non-detoxified SH as a carbon source for energy metabolism and ABE production, such that greater than 57 g/L glucose was utilized in 84 h, the *C. beijerinckii*_wildtype was not efficient in utilizing glucose in non-detoxified SH (Table 3). Consequently, approximately 25 g/L glucose was utilized by *C. beijerinckii*_wildtype during the same period. This translates into a 2.3-fold greater glucose utilization by *C. beijerinckii*_SDR when compared to *C. beijerinckii*_wildtype. For the SH detoxified with activated carbon, detoxification did not translate to improved sugar utilization by *C. beijerinckii*_SDR. However, there was improved glucose utilization by *C. beijerinckii*_wildtype leading to an increase in consumed glucose from 25 (from non-detoxified) to 46 g/L (detoxified; Table 3). Interestingly, during fermentation of non-detoxified SH with (NH_4_)_2_CO_3_ supplementation, both microbial strains utilized similar quantities of glucose at a similar rate (Table 3), even though the growth of *C. beijerinckii*_SDR was greater than that of *C. beijerinckii*_wildtype (Figure 1B). Specifically, *C. beijerinckii*_SDR utilized 42.4 g/L glucose in non-detoxified SH with (NH_4_)_2_CO_3_ supplementation, resulting in 1.3-fold less glucose utilized in non-detoxified SH without (NH_4_)_2_CO_3_ supplementation. The rate of glucose utilization by *C. beijerinckii*_SDR in a non-detoxified SH medium with (NH_4_)_2_CO_3_ supplementation was similar (0.59 g/L/h) to that of *C. beijerinckii*_wildtype (Table 3). Furthermore, when SH was detoxified with activated carbon and the medium was supplemented with (NH_4_)_2_CO_3_, the overall glucose utilization by *C. beijerinckii*_SDR and *C. beijerinckii*_wildtype was also similar. *C. beijerinckii*_SDR and *C. beijerinckii*_wildtype utilized approximately 48 g/L and 47 g/L glucose, respectively, at the rates of ∼0.68 and ∼0.66 g/L/h, respectively (Table 3).

**TABLE 3:** Summary of data from the fermentation of SH by *C. beijerinckii*_SDR and *C. beijerinckii*_wildtype. Standard deviation for readings represents triplicate (*n* = 3)*.

Medium		*C. beijerinckii*_SDR	*C. beijerinckii*_wildtype
Non-detoxified SH without medium modification	Glucose consumed (g/L)	57.71 ± 0.90^a^	24.89 ± 2.30^d^
	Maximum butanol (g/L)	11.21 ± 0.60^b^	3.58 ± 0.30^f^
	Glucose utilization rate (g/L/h)	0.60 ± 0.01^d^	0.26 ± 0.10^e^
	Maximum ABE (g/L)	20.24 ± 0.8^a^	8.98 ± 0.47^g^
	ABE yield (g/g)	0.35 ± 0.00^b^	0.36 ± 0.01^a^
	ABE productivity (g/L/h)	0.24 ± 0.00^e^	0.15 ± 0.00^g^
Non-detoxified SH with medium modification	Glucose consumed (g/L)	42.44 ± 1.20^c^	41.48 ± 0.70^c^
	Maximum butanol (g/L)	9.50 ± 0.59^e^	9.19 ± 0.75^e^
	Glucose utilization rate (g/L/h)	0.59 ± 0.01^d^	0.58 ± 0.02^d^
	Maximum ABE (g/L)	14.2 ± 0.6^e^	13.5 ± 0.80^f^
	ABE yield (g/g)	0.34 ± 0.00^b^	0.36 ± 0.01^a^
	ABE productivity (g/L/h)	0.30 ± 0.00^d^	0.37 ± 0.01^a^
Detoxified SH without medium modification	Glucose consumed (g/L)	57.86 ± 0.60^a^	45.9 ± 1.96^b^
	Maximum butanol (g/L)	12.32 ± 0.17^a^	11.05 ± 1.23^b^
	Glucose utilization rate (g/L/h)	0.80 ± 0.01^a^	0.64 ± 0.02^c^
	Maximum ABE (g/L)	17.86 ± 0.25^b^	15.09 ± 1.5 ^d^
	ABE yield (g/g)	0.32 ± 0.00^c^	0.33 ± 0.02^bc^
	ABE productivity (g/L/h)	0.37 ± 0.00^a^	0.21 ± 0.00^f^
Detoxified SH with medium modification	Glucose consumed (g/L)	48.35 ± 0.80^b^	47.2 ± 1.20^b^
	Maximum butanol (g/L)	10.72 ± 0.07^c^	10.25 ± 0.28^d^
	Glucose utilization rate (g/L/h)	0.68 ± 0.00^b^	0.66 ± 0.00^bc^
	Maximum ABE (g/L)	17.13 ± 0.34^c^	15.57 ± 0.60^d^
	ABE yield (g/g)	0.37 ± 0.00^a^	0.36 ± 0.00^a^
	ABE productivity (g/L/h)	0.36 ± 0.00^b^	0.32 ± 0.00^c^

*Statistical analysis was carried out to assess the significant difference between *C. beijerinckii*_SDR and *C. beijerinckii*_wildtype and the different medium compositions for each parameter. Same letter superscripts represent no significant differences between *C. beijerinckii*_SDR and *C. beijerinckii*_wildtype and across the different medium compositions.

#### 3.2.4 Acetone–Butanol–Ethanol Production in Switchgrass Hydrolysates

**FIGURE 5:**
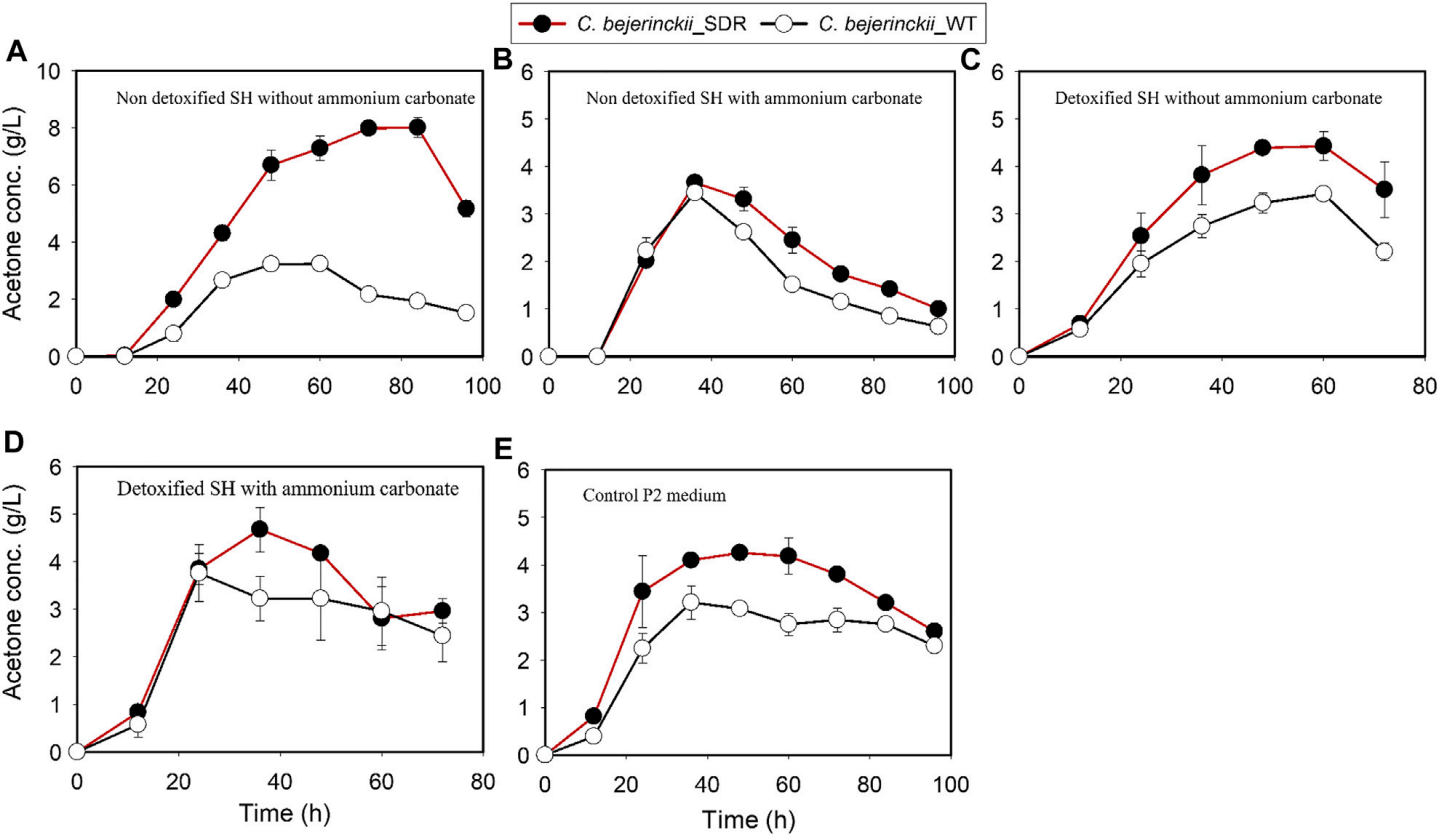
Acetone concentration profiles in cultures of *C. beijerinckii*_SDR or *C. beijerinckii*_wildtype grown in SH and P2 medium control. **(A)** Non-detoxified SH without ammonium carbonate. **(B)** Non-detoxified SH with ammonium carbonate. **(C)** Detoxified SH without ammonium carbonate. **(D)** Detoxified SH with ammonium carbonate. **(E)** Control P2 medium.

**FIGURE 6:**
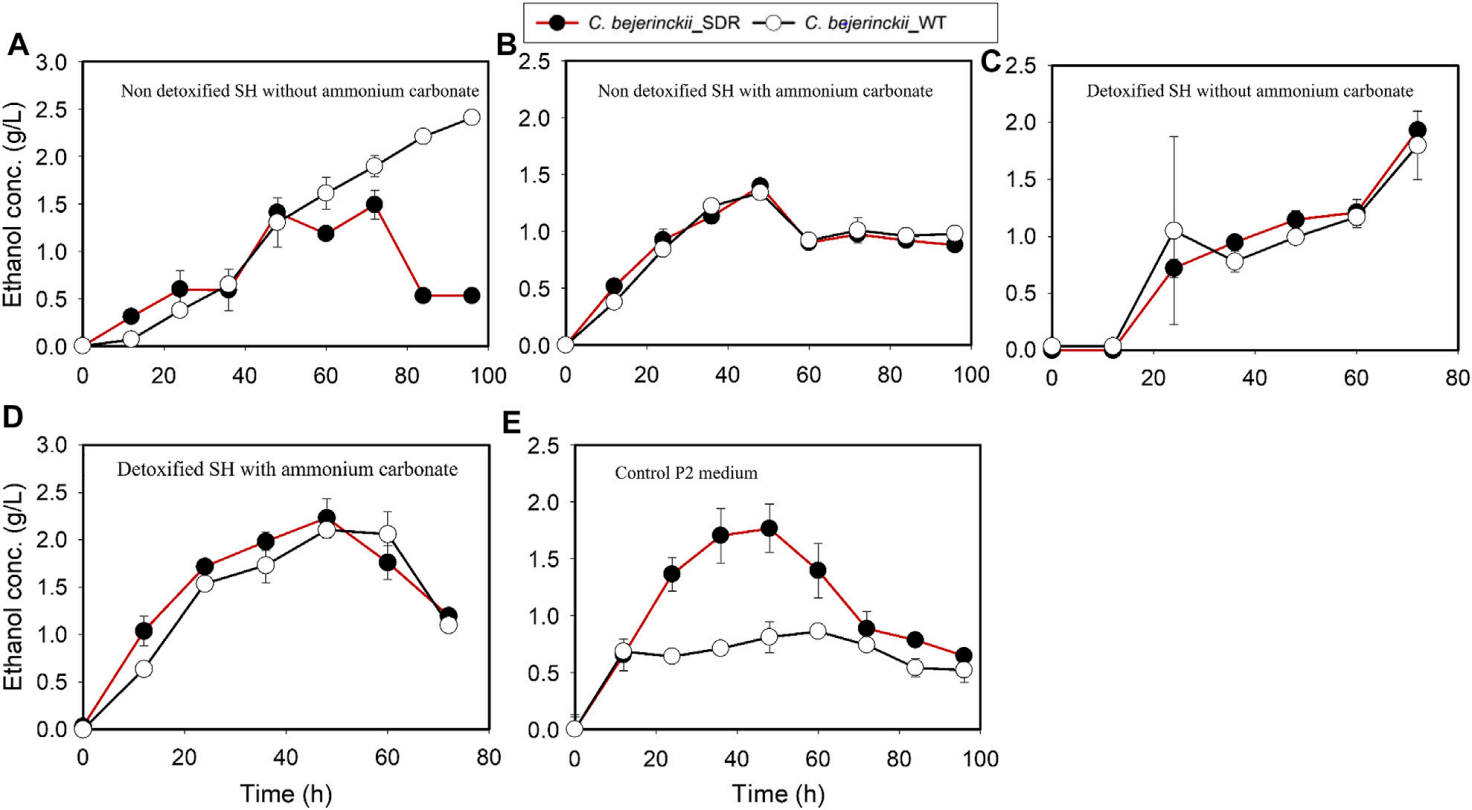
Ethanol concentration profiles in cultures of *C. beijerinckii*_SDR or *C. beijerinckii*_wildtype grown in SH and P2 medium control. **(A)** Non-detoxified SH without ammonium carbonate. **(B)** Non-detoxified SH with ammonium carbonate. **(C)** Detoxified SH without ammonium carbonate. **(D)** Detoxified SH with ammonium carbonate. **(E)** Control P2 medium.

*C. beijerinckii*_SDR produced more total ABE than *C. beijerinckii*_wildtype in all the media (non-detoxified SH, non-detoxified SH with (NH_4_)_2_CO_3_ supplementation, detoxified SH, and detoxified SH with (NH_4_)_2_CO_3_ supplementation). Notably, the quantities of acetone produced by *C. beijerinckii*_SDR were greater than that produced by *C. beijerinckii*_wildtype. Acetone concentration in the medium peaked at 8.0 g/L during fermentation of non-detoxified SH without (NH_4_)_2_CO_3_ supplementation by *C. beijerinckii*_SDR, which was 2.5-fold greater than that (3.24 g/L) produced by *C. beijerinckii*_wildtype (Figure 5A). When the non-detoxified SH medium was supplemented with (NH_4_)_2_CO_3_, the concentration of acetone in the medium was similar for both fermentations with *C. beijerinckii*_SDR and *C. beijerinckii*_wildtype (Figure 5B). Likewise, during fermentation of detoxified SH or detoxified SH with (NH_4_)_2_CO_3_ supplementation, there were greater acetone concentrations in the medium inoculated with *C. beijerinckii*_SDR than in *C. beijerinckii*_wildtype cultures. With *C. beijerinckii*_SDR, acetone concentrations were 4.4 g/L (Figure 5D) and 4.7 g/L (Figure 5C) when the SH was detoxified with and without (NH_4_)_2_CO_3_ supplementation, respectively. With *C. beijerinckii*_wildtype, acetone concentrations in the detoxified SH with and without (NH_4_)_2_CO_3_ supplementation media were 3.4 g/L (Figure 5D) and 3.8 g/L (Figure 5C), respectively. There was a marked difference in ethanol production during fermentation in non-detoxified SH without (NH_4_)_2_CO_3_ supplementation and in P2 medium (Figures 6A,E). During fermentation of non-detoxified SH without (NH_4_)_2_CO_3_ supplementation, both strains of *C. beijerinckii* exhibited similar ethanol profiles until 48 h. At the 60-h time point in cultures of *C. beijerinckii*_SDR, there was a decrease in ethanol concentration and subsequently, an increase at 72 h, while in cultures of *C. beijerinckii*_wildtype there was a sustained increase in ethanol concentration up to 96 h of fermentation (Figure 6A). The highest ethanol concentration observed with *C. beijerinckii*_SDR was 1.48 g/L at 72 h, while *C. beijerinckii*_wildtype produced 2.5 g/L at 96 h. Notably, fermentation with *C. beijerinckii*_SDR in the P2 control medium led to greater quantities of ethanol production than was observed during fermentation with *C. beijerinckii*_wildtype in the same conditions (Figure 6E). The ethanol production profiles of *C. beijerinckii*_wildtype and *C. beijerinckii*_SDR during fermentation of non-detoxified SH with (NH_4_)_2_CO_3_ supplementation and detoxified SH with (NH_4_)_2_CO_3_ supplementation were similar (Figures 6B,D).

**FIGURE 7:**
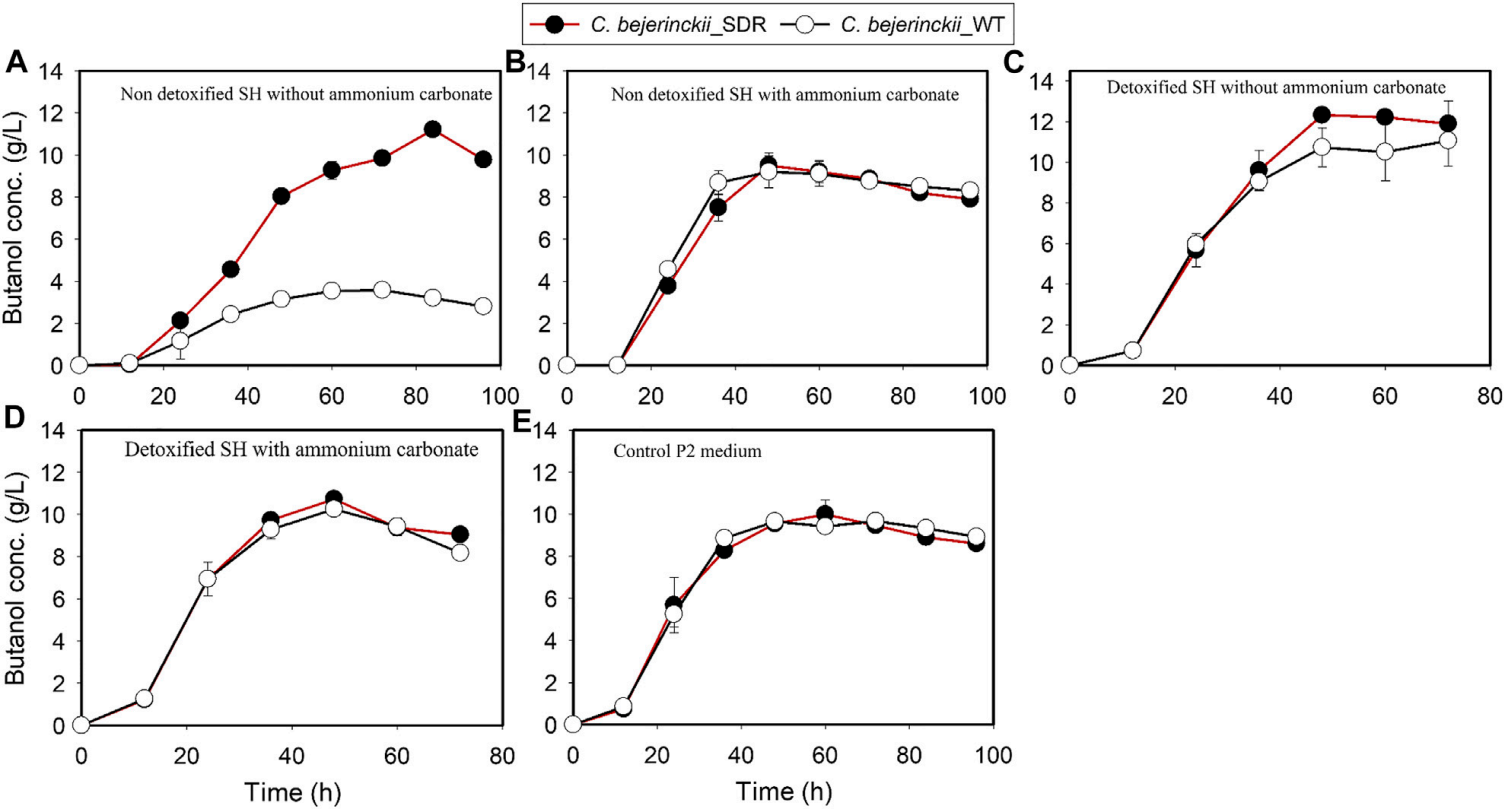
Butanol concentration profiles in cultures of *C. beijerinckii*_SDR or *C. beijerinckii*_wildtype grown in SH and P2 medium control. **(A)** Non-detoxified SH without ammonium carbonate. **(B)** Non-detoxified SH with ammonium carbonate. **(C)** Detoxified SH without ammonium carbonate. **(D)** Detoxified SH with ammonium carbonate. **(E)** Control P2 medium.

**FIGURE 8:**
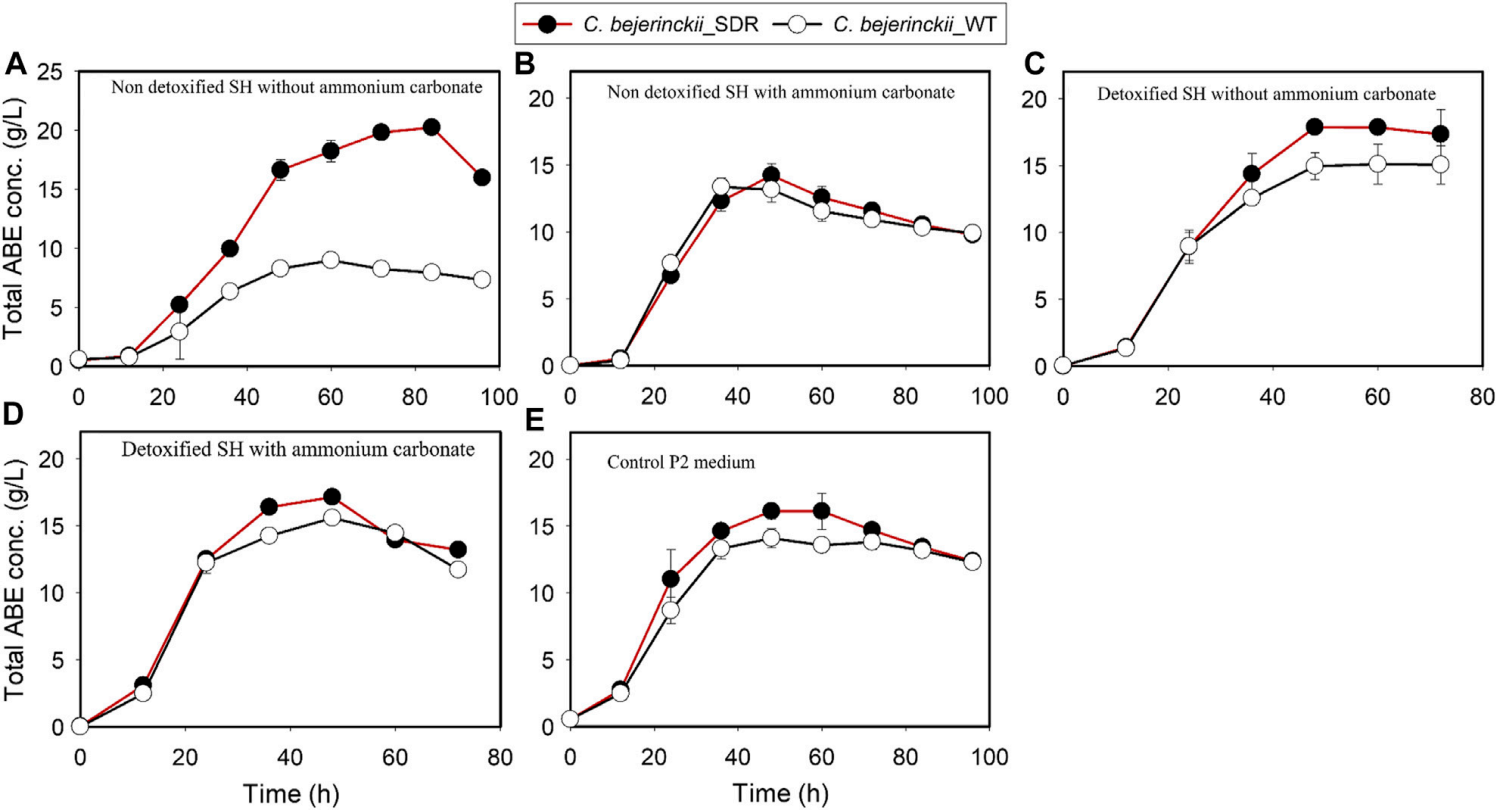
ABE concentrations in cultures of *C. beijerinckii*_SDR and *C. beijerinckii*_wildtype grown in SH and P2 medium control. **(A)** Non-detoxified SH without ammonium carbonate. **(B)** Non-detoxified SH with ammonium carbonate. **(C)** Detoxified SH without ammonium carbonate. **(D)** Detoxified SH with ammonium carbonate. **(E)** Control P2 medium.

Fermentation with *C. beijerinckii*_SDR led to 11.2 g/L and 20.2 g/L butanol and ABE, respectively (at 84 h of fermentation), when the substrate was non-detoxified SH with no (NH_4_)_2_CO_3_ supplementation, which is 3.1- and 2.2-fold greater than the maximum butanol and ABE concentrations observed with *C. beijerinckii*_wildtype (Figures 7A, 8A; Table 3). Although the 20.2 g/L ABE produced by *C. beijerinckii*_SDR in non-detoxified SH medium is greater than that it produced in P2 medium control (16.1 g/L ABE), the associated ABE productivity of 0.24 g/L/h (Table 3) was 1.5-fold less than that of the P2 medium control where the ABE productivity was 0.36 g/L/h. The relatively poor ABE productivity performance was because, while it took *C. beijerinckii*_SDR 84 h to produce a maximum ABE of 20.23 g/L in non-detoxified SH medium without (NH_4_)_2_CO_3_ supplementation, it took *C. beijerinckii*_SDR only 48 h to achieve maximum ABE concentration of 16.1 g/L in P2 medium control. When the non-detoxified SH medium was supplemented with (NH_4_)_2_CO_3_, however, *C. beijerinckii*_SDR produced 9.5 g/L butanol and 14.2 g/L ABE, which are 1.03- and 1.06- fold greater than the butanol (9.2 g/L) and ABE (13.35 g/L) produced by *C. beijerinckii*_wildtype (Figures 7B, 8B; Table 3, respectively). Interestingly, without (NH_4_)_2_CO_3_ supplementation in non-detoxified SH medium, *C. beijerinckii*_SDR produced 1.2- and 1.42-fold greater butanol and ABE (Figures 7A, 8A; Table 3), respectively, than it produced with (NH_4_)_2_CO_3_ supplementation.

To evaluate the effect of SH detoxification by activated carbon on butanol and ABE production, butanol and ABE concentrations were assessed during fermentation in detoxified SH with and without (NH_4_)_2_CO_3_ supplementation. While *C. beijerinckii*_wildtype produced 11.05 and 15.09 g/L butanol and ABE, respectively, during fermentation of detoxified SH with no (NH_4_)_2_CO_3_ supplementation, *C. beijerinckii*_SDR produced 12.32 and 17.86 g/L butanol and ABE, respectively, which were 1.11- and 1.18-fold, respectively, greater than the concentrations produced with *C. beijerinckii*_wildtype (Table 3). When the detoxified SH medium was supplemented with (NH_4_)_2_CO_3_, *C. beijerinckii*_SDR produced 17.13 and 10.73 g/L ABE and butanol, respectively, which were 1.1- and 1.05-fold greater than the respective concentrations produced with *C. beijerinckii*_wildtype (15.57 g/L ABE and 10.25 g/L butanol). Furthermore, *C. beijerinckii*_SDR produced 9.99 and 16.1 g/L butanol and ABE, respectively, while *C. beijerinckii_*wildtype produced 9.66 and 14.08 g/L butanol and ABE, respectively, during the fermentation of the control P2 medium (Figure 7E and Figure 8E), which appear to validate the greater efficacy of *C. beijerinckii*_SDR as compared with *C. beijerinckii*_wildtype to produce ABE.

## 4 Discussion

Overcoming the challenges associated with the production of butanol and ABE, particularly overcoming the hurdles posed by LDMICs has dominated research efforts during the past decade (Sarangi and Nanda, 2018; Sodre, et al., 2021). Consequently, the goal of the present study was to evaluate the capacity of *C. beijerinckii* strain constitutively overexpressing a short-chain dehydrogenase/reductase (SDR; *Cbei_*3904) to convert non-detoxified SH to butanol and ABE. Additionally, we assessed whether modification of the fermentation medium by (NH_4_)_2_CO_3_ supplementation results in a greater metabolism of the SH-derived glucose to butanol. The SDR superfamily of proteins consists of NAD(P)H-dependent oxidoreductases involved in a single-step reduction of aldehydes such as furans to less toxic alcohols (Zhang et al., 2015). Overexpression of SDR genes in non-*Clostridium* species has led to improved detoxification, hence greater tolerance to furans by these microorganisms (Almeida et al., 2008; Wang et al., 2013; Chung et al., 2015; Kim et al., 2017). Although the results from various studies indicate that concentrations as high as 2.0 g/L furfural enhances growth and solvent production in *C. beijerinckii*-wildtype (Ezeji et al., 2007b), larger concentrations of furans and other LDMICs, especially phenolic compounds (such as vanillin, p-coumaric acid, syringic acid, and hydroxybenzaldehyde, etc., even at small concentrations), are inhibitory to microorganisms involved in the production of butanol and ABE. The presence of phenolic LDMICs alongside furans in hydrolysates produced after pretreatment and hydrolysis of LB, therefore, is a barrier to effective bioconversion LB hydrolysates to fuels and chemicals.

There were 2.3-, 2.2- and 3.1-fold greater cell population, total ABE, and butanol concentrations, respectively, by the *C. beijerinckii*_SDR compared to the *C. beijerinckii_*wildtype during the fermentation of non-detoxified SH without (NH_4_)_2_CO_3_ supplementation. The lower cell density in cultures of *C. beijerinckii*_wildtype is an indication that there was marked inhibition of growth by the LDMICs present in the undetoxified SH. This contrasted with the profiles of *C. beijerinckii*_SDR grown in undetoxified SH (Table 3). The extent of inhibition observed for *C. beijerinckii*_wildtype during the fermentation of non-detoxified SH is not in agreement with the levels of LDMICs present in the SH (Table 2). This is because *C. beijerinckii*_wildtype can effectively tolerate such levels of LDMICs (Table 2) during growth and fermentation, and can detoxify up to 20-, 16-, 4-, and 2-mM furfural, HMF, 4-hydroxybenzaldehyde, and p-coumaric acid, respectively (Ezeji et al., 2007a, b; Zhang and Ezeji, 2014). It is possible that the hydrothermolysis pretreatment method used in the pretreatment of switchgrass for this study may have generated additional LDMICs that could not be detected by the HPLC-based analytical procedure described by Agu et al. (2016). Nonetheless, the results from the present study provide further evidence that *C. beijerinckii_*SDR can detoxify or tolerate LDMICs at the concentrations present in the SH, as evidenced by the increased cell population during fermentation (Figure 1A). This is further evidenced by the effective conversion of SH-derived glucose to butanol and ABE during fermentation of undetoxified SH by *C. beijerinckii_*SDR (Figures 7A, 8A). Although the robustness of *C. beijerinckii_*SDR in terms of fermentation efficacy was highlighted during the fermentation of non-detoxified SH, the fermentation profile of this strain (butanol and ABE production) during fermentation of non-detoxified SH with (NH_4_)_2_CO_3_ supplementation further underscored its potential.

Comparing the growth patterns of *C. beijerinckii_*SDR and *C. beijerinckii_*wildtype in non-detoxified SH, with and without (NH_4_)_2_CO_3_ supplementation alongside the acid production profiles, the inhibitory actions of LDMICs (Table 2) appeared to be compounded by the acetic acid content of the SH (Figure 3). Acetic acid concentrations at 0 h in fermentations with *C. beijerinckii_*SDR (9.74 g/L) or *C. beijerinckii_*wildtype (7.92 g/L) were greater than the acetic acid concentrations (∼3–4 g/L) typically present at the initiation time point of ABE fermentation. These relatively lesser acetic acid concentrations at 0 h are desired to maintain a pH range that supports microbial population growth and ABE production. In the present study, the acetic acid produced during pretreatment of switchgrass and that from the buffer component of the P2 medium resulted in a greater acetic acid concentration of the SH fermentation medium at 0 h (Figure 3). Because ABE fermentation with *C. beijerinckii* is bi-phasic, acetic acid produced during the acidogenic/microbial population growth phase exacerbated the considerably greater acetic acid concentration of the fermentation medium. Consequently, there were two types of adaptive challenges during the fermentation period for *C. beijerinckii_*SDR and *C. beijerinckii_*wildtype. The higher acid concentration in the fermentation medium appears to be the major inhibitory factor to the growth and survival of *C. beijerinckii*_wildtype and *C. beijerinckii*_SDR during fermentation. Lower concentrations of protonated acetic and butyric acids and greater buffering capacity provided because of supplementation with (NH_4_)_2_CO_3_ may have contributed to the enhanced growth observed for *C. beijerinckii_*SDR and *C. beijerinckii_*wildtype during fermentation of non-detoxified SH (Figure 1B).

While these larger cell populations led to 2.6- and 1.5-fold increase in butanol and ABE production, respectively, for *C. beijerinckii_*wildtype, supplementation of the non-detoxified SH medium with (NH_4_)_2_CO_3_ had a negative effect on ABE production in cultures inoculated with *C. beijerinckii_*SDR. Consequently, there was 1.2- and 1.4-fold less butanol and ABE production (Table 3), respectively, during the growth of *C. beijerinckii_*SDR in non-detoxified SH with (NH_4_)_2_CO_3_ supplementation than in non-detoxified SH medium without (NH_4_)_2_CO_3_ supplementation (Table 3). These findings are consistent with the previous findings of Han et al., (2013) where supplementation of fermentation medium with carbonates resulted in greater buffering of the medium and larger cell populations of the microorganism under investigation. While supplementation of the fermentation medium with CaCO_3_ at ≥4 g/L resulted in a marked increase in ABE production, there was only a slight increase in total ABE production with (NH_4_)_2_CO_3_ supplementation (Han et al., 2013). For ABE fermentation using *Clostridium* species, pH is a major indicator of the acidogenic growth phase and its effects on ABE production (Gottwald and Gottschalk, 1985). It should be noted that the association between the magnitude of cell population and ABE production is not always linear (Han et al., 2013). During the solventogenic phase of the fermentation which is characterized by ABE production, maintenance of the medium pH in the range of 5.0–5.5 is optimal for ABE production while a higher pH typically leads to acid accumulation (Bahadur and Saroj, 1960; Bahl et al., 1982; Monot et al., 1984; Wu et al., 2017). The pH range of the medium during the solventogenic phase was 5.2–5.6 in cultures of *C. beijerinckii_*SDR grown in non-detoxified SH without (NH_4_)_2_CO_3_ supplementation. These conditions resulted in the maximum ABE production of 20.24 g/L (Table 3). Conversely, *C. beijerinckii*_SDR maintained pH above 5.5 in the non-detoxified SH medium supplemented with (NH_4_)_2_CO_3_ during solventogenic phase of the fermentation (Figure 2B). It is possible, therefore, that the buffering effect of (NH_4_)_2_CO_3_ resulted in a sustained pH greater than 5.5, which led to enhanced growth of *C. beijerinckii*_SDR that was, in turn, sub-optimal for ABE production. Hence, there was a lesser ABE production with *C. beijerinckii*_SDR in non-detoxified SH medium with (NH_4_)_2_CO_3_ supplementation.

As expected, both *C. beijerinckii_*SDR and *C. beijerinckii_*wildtype produced higher butanol and ABE concentrations in detoxified SH without (NH_4_)_2_CO_3_ supplementation than in the P2 medium (Figure 8; Table 3). The improvement was because the LDMICs present in the SH, that was pretreated using hydrothermolysis procedures had been markedly reduced by the activated carbon detoxification treatment. Consequently, the growth of *C. beijerinckii_*SDR in the detoxified SH was rapid, leading to high glucose consumption thereby, achieving a maximum ABE of 17.86 g/L in 48 h compared to a maximum ABE of 15.09 g/L in 72 h for the *C. beijerinckii_*wildtype. Furthermore, the ABE productivity of 0.37 g/L/h achieved with *C. beijerinckii_*SDR was greater than the ABE productivity of 0.27 g/L/h observed for *C. beijerinckii_*wildtype (Table 3). The ABE yield with both *C. beijerinckii_*wildtype and *C. beijerinckii_*SDR varied between 0.32 and 0.37 g ABE/g glucose during fermentation of SH or P2 medium control. There was no trend or pattern of ABE production that was indicative of factors or conditions that affected ABE yield. It is possible that some carbons were diverted to the production of compounds such as formic and lactic acid as these compounds are typically produced relatively in marked amounts by solventogenic *Clostridium* species under unfavorable growth conditions.

The capacity of *C. beijerinckii*_SDR to increase in population size and ferment non-detoxified SH relative to that of *C. beijerinckii*_wildtype in substrates replete with LDMICs and high levels of acetic acid indicates that overexpression of the SDR (*Cbei_*3904) in *C. beijerinckii* conferred some resiliency on the strain, in conditions that were inhibitory to *C. beijerinckii*_wildtype. Although the protein product of *Cbei_*3904 has been re-annotated as a tri/tetra-hydroxynaphthalene reductase-like enzyme, it is still an oxidoreductase and SDR superfamily domains (https://www.ncbi.nlm.nih.gov/protein/WP_012060066.1; https://www.kegg.jp/dbget-bin/www_bget?cbe:Cbei_1071+cbe:Cbei_2398+cbe:Cbei_3904). The tri/tetra-hydroxynaphthalene reductases are specifically involved in fatty acid biosynthesis, co-factor (biotin) metabolism, and reduction of alternate phenolic compounds and cyclic ketones in bacteria (Schätzle et al., 2012; Okonkwo et al., 2019). This background information leads to the suggestion that the protein product of *Cbei_*3904 is possibly involved in modulating the lipid composition of the cell membrane of *C. beijerinckii*_SDR and consequently, fortifying membrane integrity, thus, improving tolerance to LDMICs. Interestingly, modulation of lipid biosynthesis and composition occurs in *Saccharomyces cerevisiae* in response to the presence of organic acids and phenolic compounds during fermentation of LB hydrolysates (Guo et al., 2018). Overexpression of the *OLE1* gene that encodes a protein responsible for the synthesis of monounsaturated fatty acids in *S. cerevisiae* leads to an increased monounsaturated fatty acid content of the plasma membrane and conferment of enhanced tolerance to the deleterious effects of acetic acid and phenolic compounds (Guo et al., 2018). Considering the tolerance of *C. beijerinckii*_SDR to the LDMICs in non-detoxified to SH and the capacity to grow and convert glucose ABE in SH, it is likely that a similar mechanism (as in *S. cerevisiae*; Guo et al., 2018) might account for the robust capacity of *C. beijerinckii*_SDR to grow in and ferment non-detoxified SH to ABE.

The large quantities of acetone produced by *C. beijerinckii*_SDR in comparison to *C. beijerinckii*_wildtype during fermentation in all the media evaluated in the present study including in the P2 medium control (Figure 5) are noteworthy. This may be due to greater utilization of NAD(P)H because of the insertion of an additional copy of SDR gene in *C. beijerinckii* and hence reduced availability of NAD(P)H for butanol production. Integration of the *Cbei_*3904 into the genome of *C. beijerinckii* under the control of a constitutive promoter (thiolase) ensures continued expression of the associated enzyme during both the acidogenic and solventogenic phases of growth (Okonkwo et al., 2019). As a NAD(P)H-dependent oxidoreductase, continued expression of *Cbei_*3904, which likely promotes fatty acid biosynthesis, co-factor (biotin) metabolism, and NAD(P)H-consuming reduction of phenolic compounds (Schätzle et al., 2012; Okonkwo et al., 2019), may be in direct competition with butanol production, which is also NAD(P)H-dependent. A limiting quantity of NAD(P)H in the cytoplasm of *C. beijerinckii*, even for a short period, can have ramifications that include accumulation and decarboxylation of acetoacetic acid to acetone and CO_2_ (Han et al., 2011), and increased growth of *C. beijerinckii* (Figure 1) due to the abundance of NAD^+^ for glycolysis (Zhang et al., 2012; Ujor et al., 2014). In the presence of LDMICs (Figures 5A,C) and to sustain cell population, active detoxification of LDMICs by *C. beijerinckii* requires repartitioning of NAD(P)H utilization for different processes, with potential beneficial effect on non-NAD(P)H-dependent acetone production, which facilitates the ability of the cell to reabsorb and convert acetic acid to a neutral product (acetone).

## 5 Conclusion

In the present study, the capacity of a genetically engineered strain of *C. beijerinckii* NCIMB 8052 (*C. beijerinckii_*SDR) to tolerate the deleterious effects of LDMICs and produce ABE with hydrothermolysis-pretreated SH was evaluated. Additionally, the effect of fermentation medium modification by (NH_4_)_2_CO_3_ supplementation on the fermentation profile of *C. beijerinckii_*SDR was investigated. Supplementation of SH with (NH_4_)_2_CO_3_ led to improved growth of *C. beijerinckii* strains and ABE production. Use of non-detoxified SH along with metabolically engineered *C. beijerinckii*_SDR to produce ABE is a promising combination as it produced 1.26-fold (20.24 g/L ABE) more ABE than the positive control (16.1 g/L ABE). This result underscores the robustness of *C. beijerinckii*_SDR for ABE production using undetoxified hydrothermolysis-pretreated SH. Moreover, our results make a case for metabolic engineering as a tool for rewiring metabolic networks in fermenting microorganisms toward enhanced production of fuels and chemicals using cheap biomass substrates.

## Data Availability

The original contributions presented in the study are included in the article/Supplementary Material; further inquiries can be directed to the corresponding author.
